# Preterm Infants Without Neurological Damage and Nursery Care: Risks, Benefits and Prospects for Intervention

**DOI:** 10.3390/children12091162

**Published:** 2025-08-31

**Authors:** Micaela Capobianco

**Affiliations:** Economic, Psychological and Communication Sciences Department, Niccolò Cusano University, 00166 Rome, Italy; micaela.capobianco@unicusano.it

**Keywords:** preterms without neurological damage, nursery, specific disorders, prevention, multifactorial developmental model

## Abstract

Background/Objectives: Preterm infants without neurological damage are at greater risk of developing specific difficulties due to the unpredictable effects of the interaction between biological immaturity and characteristics of physical and social environment. This paper discusses the potential advantages and limitations of placing premature infants in daycare in the first three years. Methods: In light of the scientific literature, the article discuss-es the topic through a critical comparison of studies on the developmental outcomes of preterm infants, on the characteristics of nurseries and on the role of educators. Results: Nursery placement must be evaluated in an integrated and multifactorial perspective, taking into account the specific vulnerabilities of each individual preterm child and the characteristics of the nursery. The role of educators is to structure a quality educational environment that meets the specific needs of each individual preterm child, in close collaboration with parents and specialists. Conclusion: This article provides suggestions to parents, educators, and specialists for the assessment and intervention with preterm children without neurological impairment in the educational setting, with a multidisciplinary view to prevention and early intervention.

## 1. Introduction

Children born preterm *without neurological damage* (here in after only “preterm”) are born before the 37th week of gestational age, without clinically detectable brain damage [1,2]. In the clinical and research fields, there are still many debates regarding the methodology for assessing the developmental risk of preterm infants.

The World Health Organization (WHO) considers prematurity based on two parameters: gestational age and birth weight, considered—until 1949—as independent criteria in the evaluation of preterm newborns. Subsequently (from the second half of the 1970s), it was possible to construct curves corresponding to the average prenatal growth values and differentiate preterm newborns based on the relationship between weight and gestational age [3,4]. Currently, preterm children are divided in a. small for gestational age (SGA); b. large for gestational age (LGA); c. appropriate for gestational age (AGA). The evaluation of “adequacy or otherwise” between gestational age and weight constitutes a specific parameter, important for a more refined definition of the biological risk of a preterm infant [5], but this parameter is not sufficient to define the developmental risk of each preterm child [3,6]. An infant preterm is at greater risk the lower the gestational age, which leads to greater brain immaturity, but the condition of severity can increase due to additional associated medical problems and influence developmental outcomes in the first three years of life [2,5,6]. Clinical and research evidence suggests that preterm children are particularly at risk of developing specific developmental disorders (for example: Learning and Language disorder, attention disorder) due to the impact of “brain immaturity” (the lower the gestational age) with the characteristics of the physical and social environment. Most preterm children fully recover from their initial disadvantage by about two to three years of age, but for some, the developmental process proceeds toward a consolidation of the initial delay [1,5,7,8,9,10]. The literature agrees on a multifactorial hypothesis, according to which other factors (primarily environmental) may play an important role in the recovery process of these children, starting from very different biological risk conditions [4]. The initial state of biological vulnerability, in dynamic interaction with other factors (protective or of risk), can favor or hinder the natural recovery process of the premature child.

Within this theoretical framework, the “kangaroo care” can be an important experience for preterm children, fostering their socio-cognitive and communicative-linguistic development through social experiences with peers and adults. At the same time, the “kangaroo care” requires special attention for these children, given their greater initial vulnerability and the interaction between environmental and biological factors, the course of which is still unpredictable [11,12].

This article aims to critically discuss the advantages and limitations of nursery placement for preterm children, in relation to the characteristics of the educational context and the profile of these children, from a multifactorial perspective and for prevention and early intervention within the first three years of age [13,14]. A critical discussion- in an opinion article-on this topic can be useful to parents, educators and specialists in a perspective of continuous and dynamic sharing and collaboration, with respect to the choice of the age of inclusion and the organization of spaces, activities and intervention strategies to be structured in the context of the nursery according to the specific profile and individual needs of each preterm child [15].

## 2. Advantages and Limitations of the Educational Context: Comparing Literature

The educational context offers behavioral models for learning emotional regulation and building socio-affective skills [16]. Some studies show that attendance at quality educational services in nursery can positively impact language development and cognitive abilities [2,5]. This effect is particularly evident in children from disadvantaged socioeconomic backgrounds and at developmental risk, for whom nursery school can represent an important opportunity to compensate and reduce inequalities [17,18].

Interactions with peers and adults other than parents contribute to the development of communication and symbolic skills, fostering the formation of personal and social identity. Although emotions have a biological basis, the way they are experienced and interpreted is profoundly influenced by the educational and relational environment [2,5,8,15]. In a longitudinal study with preterm children [9], observed that participation in high-quality educational programs in the early years of life is associated with better cognitive outcomes at age five. The stimulating environment of daycare could therefore represent a protective factor, helping to mitigate any delays in early development related to prematurity and promote the natural recovery process. One of the strengths of the daycare experience is the opportunity to interact daily with a group of peers and adults other than primary caregivers. In this context, children learn to share, collaborate, manage conflict, and recognize emotions—both their own and those of others.

Dalli et al. [19] confirm that regular nursery experience fosters the development of relational and cooperative skills. For preterm children, who may have greater difficulties with emotional and behavioral regulation, these relational opportunities are particularly important.

From a family perspective, being able to entrust their preterm child to a safe and professional environment provides important support in managing work and family life [4].

However, there are also critical issues to consider.

One of the main concerns is the increased risk of infections, particularly respiratory and gastrointestinal infections. Several studies confirm that children who attend nursery tend to get sick more frequently, especially in the first year of attendance [20]. This problem is particularly relevant for premature babies, who are often more immunocompromised. Some studies observe that children born before 32 weeks and enrolled in nursery in the first two years of life have a significantly higher risk of hospitalization for respiratory infections compared to their peers born at term. It is therefore essential to evaluate, together with the pediatrician, the timing and modality of inclusion based on the child’s health. For some preterm children, a nursery attendance of no more than 5 h may be appropriate, and in a class with the right balance between the number of children and teachers [4].

Another delicate aspect concerns the emotional impact of separation from parents. For some younger children, between 8 and 15 months, and especially for those with a more sensitive temperament and more vulnerable emotionally and behaviorally, daily separation can be a source of stress. Some studies have found increased cortisol levels during days spent in nursery, a sign of stress activation. This study observed a greater reactivity to environmental stimuli and a heightened sensitivity, characteristics that make them less able to adapt to the complexity of nursery stimuli and less resilient [21,22]. In this perspective, Kangaroo Mother Care (KMC), known internationally as “Pouch Care,” is a scientifically validated practice that involves skin-to-skin contact between the preterm infant and the parent, both in the hospital and at home [10,23]. Recent studies show that this therapy promotes neurological and psychological recovery, stabilizes cardiac and respiratory function, and promotes breastfeeding and weight gain. The role of parents and their training is therefore fundamental in providing a quality relationship from the earliest stages of development, when preterm infants are highly vulnerable. These studies demonstrate the important role of parents in the early stages of interaction with their preterm infant, in the process of emotional regulation and the development of socio-affective and communicative-linguistic skills. The possibility of successful integration into nursery school is closely linked to the quality of early mother-infant interactions [23].

This means that for preterm children it is important to ensure gradual insertion, evaluating various factors, including: (a) the infant’s medical conditions; (b) the most suitable age for insertion; (c) the characteristics of the nursery (quality elements), including the number of children and teachers in the class. The consideration of the different factors makes it possible to guarantee an educational environment functional to the needs of each child [17]. In support of the research data, clinical experience shows that preterm children, even if free from neurological damage, are more likely to incur specific developmental disorders, emotional and behavioral dysregulation, most likely due to the complex interaction between biological immaturity and multiple environmental factors, the outcome of which is difficult to predict. The inclusion of the preterm child in the nursery could be a protective factor if organized in a functional way to the specific developmental and health characteristics of the individual child.

## 3. Quality of the Nursery and Role of the Educator: A Specific Choice

A decisive factor in determining the impact of the nursery on the development of premature children is the quality of the service. A study by [24] showed that children with experience in high-quality educational services in nursery have better emotional self-regulation when they start primary school. Not all facilities guarantee the same level of attention to care, pedagogical planning, and staff training. The ratio of educators to children, the quality of the spaces, and the variety of activities offered are crucial aspects to evaluate [18]. Research shows that while high-quality services offer significant benefits, low-quality services may have a negative impact on socio-emotional development. This is particularly relevant for preterm children, for whom the quality of the educational environment is even more crucial in promoting natural recovery in the first three years of life.

A educational environment, with meaningful relationships and appropriate stimuli, can promote cognitive, language, emotional and social development, acting as a protective factor against possible developmental difficulties [2,5].

However, it is essential that the educational context be structured with awareness of potential risks, both physical (such as exposure to infections) and the emotional impact of separation from primary attachment figures. The role of the educator in early childhood services has undergone significant evolution [12,15,25]. Previously, educators were seen primarily as caregivers. Today, they are recognized as highly qualified professionals, fully aware of their role. It is no longer simply about caring for children but about accompanying them on their growth journey with solid pedagogical skills and constantly learning through experience [26]. The educator’s work can be imagined as a system that operates on three closely related dimensions: (1) Relational, which is based on building meaningful bonds with children and their families. (2) Planning, in which observation, documentation, and planning become fundamental tools for creating tailored educational programs. (3) Organizational, which requires the ability to effectively manage space, time, and materials to offer a stimulating and welcoming environment [12,15,25]. At the heart of all this is a specific vision of the child: no longer a passive subject, but an active and competent being, with their own unique profile, capable of actively constructing their own learning, in continuous interaction with peers and competent adults. One of the key skills of the educator is the ability to work in a team, collaborating to build a shared educational project. Discussion between colleagues and specialists not only fosters professional growth but also improves the overall quality of the nursery [27].

In daily educational activities, the active involvement of parents is also very important. From a co-education perspective, it is essential to build an educational alliance with parents, based on dialog, mutual respect and shared responsibilities [24]. This relationship takes on particular importance during the settling-in period, when the child makes the delicate transition from home to nursery school. Furthermore, through careful and continuous observation, the educator can grasp the unique characteristics, needs, and potential of each individual preterm child. Some works highlight the importance of educators’ ability to interpret the prosody of vocalizations and crying to correctly interpret specific needs of the infant in the educational context [28,29].

Educational planning should not be understood as the rigid application of predefined frameworks, but as a flexible and dynamic process, capable of responding to emerging needs [28]. The quality planning combines pedagogical intentionality and openness to the unexpected, striking a balance between structure and freedom.

The relational dimension is a fundamental aspect: connecting with children, correctly interpreting their communicative signals, and creating a positive emotional climate is crucial to their well-being and harmonious development [17].

Educators also play a central role in promoting inclusion. In line with the most current pedagogical perspectives, they are called upon to build educational contexts that welcome and value diversity, ensuring equal growth opportunities for all children [20]. This means constantly adapting tools, strategies, and educational approaches to the specific needs of each child [15,30]. Inclusive educators are able to understand educational needs in their entirety, recognizing not only the difficulties but also the resources of each child. As Pavone [31] argues, this involves overcoming a deficit-based approach to embrace a perspective centered on the value of differences. This change translates into practices oriented towards the active and meaningful participation of all respecting individual differences [20,24,32].

## 4. Conclusions

The current manuscript discusses in modo critic the advantages and limitations of nursery placement for preterm infants *without neurological damage*, considering the biological vulnerability of these children and the characteristics of the nursery. The work aims to provide specific information to parents, educators and specialists, through a critical analysis of research and clinical data with respect to the possibility and methods of inclusion in the nursery for children born prematurely without neurological damage. Placing preterm infants in daycare can have advantages and limitations, depending on the interaction between the specific needs of the premature infant and the quality of the educational environment. Welcoming parents of preterm infants requires specific skills and targeted training [30]. Preterm infants may exhibit atypical neuromotor, cognitive, linguistic, and socio-emotional developmental trajectories, requiring tailored educational interventions [24]. In this context, the educator plays an important mediating role between the child, the family, and healthcare professionals, acting as a bridge between the different living environments and creating a true “developmental safety net” [11].

When working with premature infants, careful observation and individualized planning become even more important given their risk. Targeted educational interventions, based on careful observation and structuring of activities in the educational context, can contribute to addressing learning delays, particularly in the communicative and social spheres [33]. Another dimension of the educational role concerns parenting support. Parents of preterm infants may experience feelings of anxiety, insecurity, and guilt. Educators, through empathetic listening and a collaborative relationship, can help them recognize their child’s potential and strengthen their parental trust. The physical organization of the nursery environment is crucial in ensuring adequate responses to the specific needs of preterm infants. Recent research highlights how carefully modulating the timing and stimuli present in the educational space, such as sounds, lights, and materials, can significantly contribute to the emotional stability and active engagement of these children, facilitating their integration and participation in educational programs [25,28].

Only in this way can we ensure an inclusive approach, capable of responding sensitively and competently to the unique characteristics of each child and each family, making the nursery a place of well-being and shared growth, and enhancing natural recovery in the first three years of life [32,34,35].

The training of educators becomes a fundamental element, and concerns diversified knowledge: on the biological vulnerability of the preterm infant, on relational and communicative strategies with children and parents, on activities to promote neuropsychological development, on the organization of spaces and functional objects for each preterm child [15,35]. The choice of the age of inclusion and the daily dwell time in the nursery is linked to: a. the assessment of the child’s medical condition; b. the psychoeducational support that the parents have had to promote interaction with the child in the hospital and at home after birth; c. the characteristics of the educational contest [10]. In some cases, it may be preferable to insert the premature child from 18-24 months of age, considering that research and clinical data show a natural recovery of developmental skills in the first two years of age and the importance of the parent–child attachment relationship during two years old [5].

In a perspective of prevention and early intervention of developmental risk, a fundamental aspect concerns the collaboration and continuous sharing between parents, school and specialists, each with his or her own role, with the common goal of building life contexts that promote the recovery process of preterm children. It can be concluded by highlighting that a multifactorial and multidisciplinary approach seems to be the most functional for the evaluation and intervention in favor of children born preterm without neurological damage.

In conclusion, the team, consisting of a paediatrician, developmental psychologist, neuropsychiatrist and educators, could assess the age of placement, the hours of stay of the preterm child, in sharing with the family, and organise the spaces and activities most functional to promote development, starting from the specific condition of vulnerability [14,15,24,32,33].

## Data Availability

No new data were created.
